# P-779. Macronutrient Status and Risk of Tuberculosis vs. Other Lower Respiratory Tract Infections

**DOI:** 10.1093/ofid/ofae631.973

**Published:** 2025-01-29

**Authors:** Joanne Wu, Peter Cegielski

**Affiliations:** Emory University, Atlanta, Georgia; Rollins School of Public Health Emory University, Atlanta, Georgia

## Abstract

**Background:**

Nutritional status is a major risk factor for incident tuberculosis disease (TB), but the underlying biological mechanisms remain unknown. If this effect is mediated immunologically, then other lower respiratory tract infections (LRTI) that elicit different immune responses would differ in this regard. If it is due to “body habitus,” then a similar association might be seen in anatomically similar infections. We compared the effect of nutritional status on TB, pneumococcal pneumonia (pneumonia), and influenza pneumonia (flu).

**Figure:**
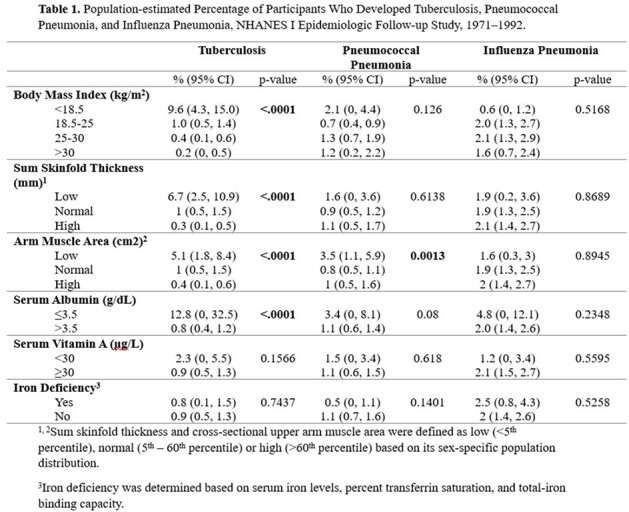

**Methods:**

The first National Health and Nutrition Examination Survey (NHANES-I) provided data on baseline nutritional status of a probability sample of the US population, 1971-1975. Nutrition indicators included body mass index (BMI), skinfold thickness (SFT), cross-sectional upper arm muscle area (AMA), serum albumin, serum vitamin A, and iron status. The NHANES-I Epidemiological Follow-up Study provided follow-up data through 1992. Incident TB, pneumonia, and flu were ascertained through ICD9 codes in medical records and death certificates. For each nutrition indicator, we compared population-estimated percentages of those who later develop TB, pneumonia, and flu.

**Results:**

An estimated 1.2% of the population developed TB: 9.6% (95% CI: 4.3, 15.0) of those with BMI < 18.5 kg/m^2^) developed TB compared with 1% (0.5, 1.4) of those with normal BMI, 18.5-25 kg/m^2^, and 0.4% (0.1, 0.6) of those with BMI 25-30 kg/m^2^ (Table 1). In contrast, BMI was not associated with developing pneumonia or flu. Like BMI, SFT and AMA were also inversely associated with incident TB but not with flu or pneumonia. In fact, pneumonia had an “U”-shaped association with AMA (Table 1). Among those with hypoalbuminemia (≤3.5 g/dL), 12.8% (0, 32.5) developed TB versus 0.8% (0.4, 1.2) of those with normal albumin >3.5 g/dL. Serum albumin was not associated with incident pneumonia or flu. Serum Vitamin A and iron deficiency were not associated with any of these three LRTI.

**Conclusion:**

These differences suggest host defenses against TB, mainly adaptive cell-mediated immune responses, are sensitive to macronutrient status while the acute granulocytic and innate humoral defenses against other LRTI pathogens are not affected in the same way.

**Disclosures:**

**All Authors**: No reported disclosures

